# No evidence for local adaptation of dengue viruses to mosquito vector populations in Thailand

**DOI:** 10.1111/eva.12360

**Published:** 2016-02-24

**Authors:** Thanyalak Fansiri, Arissara Pongsiri, Chonticha Klungthong, Alongkot Ponlawat, Butsaya Thaisomboonsuk, Richard G. Jarman, Thomas W. Scott, Louis Lambrechts

**Affiliations:** ^1^ Department of EntomologyArmed Forces Research Institute of Medical SciencesBangkokThailand; ^2^ Department of VirologyArmed Forces Research Institute of Medical SciencesBangkokThailand; ^3^ Department of Entomology and NematologyUniversity of CaliforniaDavisCAUSA; ^4^ Fogarty International CenterNational Institutes of HealthBethesdaMDUSA; ^5^ Insect‐Virus Interactions GroupDepartment of Genomes and GeneticsInstitut PasteurParisFrance; ^6^ Unité de Recherche AssociéeCentre National de la Recherche ScientifiqueParisFrance; ^7^ Present address: Viral Diseases BranchWalter Reed Army Institute of ResearchSilver SpringMDUSA

**Keywords:** *Aedes aegypti*, dengue virus, genotype × genotype interactions, local adaptation, population structure

## Abstract

Despite their epidemiological importance, the evolutionary forces that shape the spatial structure of dengue virus genetic diversity are not fully understood. Fine‐scale genetic structure of mosquito vector populations and evidence for genotype × genotype interactions between dengue viruses and their mosquito vectors are consistent with the hypothesis that the geographical distribution of dengue virus genetic diversity may reflect viral adaptation to local mosquito populations. To test this hypothesis, we measured vector competence in all sympatric and allopatric combinations of 14 low‐passage dengue virus isolates and two wild‐type populations of *Aedes aegypti* mosquitoes sampled in Bangkok and Kamphaeng Phet, two sites located about 300 km apart in Thailand. Despite significant genotype × genotype interactions, we found no evidence for superior vector competence in sympatric versus allopatric vector–virus combinations. Viral phylogenetic analysis revealed no geographical clustering of the 14 isolates, suggesting that high levels of viral migration (gene flow) in Thailand may counteract spatially heterogeneous natural selection. We conclude that it is unlikely that vector‐mediated selection is a major driver of dengue virus adaptive evolution at the regional scale that we examined. Dengue virus local adaptation to mosquito vector populations could happen, however, in places or times that we did not test, or at a different geographical scale.

## Introduction

When natural selection is spatially heterogeneous and results in genotype × environment (G × E) interactions, one can expect higher relative fitness of resident genotypes in their habitat than genotypes originating from different habitats. Local adaptation refers to the process underlying higher fitness of resident genotypes (Williams 1966) and has been the subject of considerable theoretical and experimental literature in ecology and evolutionary biology (Kawecki and Ebert 2004; Blanquart et al. 2013). This is because adaptation to local environmental conditions is central to several fundamental processes, such as maintenance of biodiversity, species distribution, and dynamics of species interactions. In addition, it provides insights into the strength of natural selection relative to gene flow and other evolutionary forces. In particular, local adaptation has received considerable attention in host–parasite systems, where G × E interactions often occur in the form of specific parasite genotype × host genotype (G × G) interactions (Kaltz and Shykoff 1998; Greischar and Koskella 2007). Theory predicts that parasite local adaptation, in which sympatric host–parasite combinations result in higher infection success than do allopatric combinations, is favored due to shorter generation times, larger population sizes, and higher migration rates of parasites than their hosts. This prediction is partially supported by results from experimental studies (Greischar and Koskella 2007).

Here, we investigated the evolutionary concept of local adaptation to better understand the spatial distribution of a major viral pathogen of humans. Dengue viruses (DENVs) are RNA viruses of the genus *Flavivirus* that cause more human disease than any other arthropod‐borne (arbo) virus (Guzman and Harris 2015). They are transmitted among human hosts by mosquito vectors, primarily *Aedes aegypti* (Lambrechts et al. 2010). They cause an estimated 390 million human infections each year, of which about a quarter are clinically apparent (Bhatt et al. 2013). Dengue symptoms range from clinically inapparent to self‐limiting fever to life‐threatening illness (Simmons et al. 2012). In the absence of a licensed vaccine or therapeutic drug, dengue prevention efforts are currently limited to vector control measures, which have proven difficult to effectively apply and even harder to sustain over a long period of time (Morrison et al. 2008).

Dengue viruses harbor extensive genetic diversity, most notably in the form of four serotypes (DENV‐1, ‐2, ‐3, and ‐4), which are phylogenetically closely related (Holmes and Twiddy 2003) and loosely antigenically distinct (Katzelnick et al. 2015). Spatially, DENV genetic diversity is often characterized by clustering at a small geographical and temporal scale (Holmes and Twiddy 2003). Previous studies on DENV microevolution in Southeast Asia indicated that spatial patterns of genetic diversity are shaped by frequent virus immigration and spatially and temporally focal transmission (Jarman et al. 2008; Raghwani et al. 2011; Rabaa et al. 2013). While there is compelling evidence for genetic differences in virulence and epidemic potential among DENV genotypes, lineages, and strains (Rico‐Hesse 2003), the evolutionary forces that shape the spatial structure of DENV genetic diversity presently are not fully understood.

Earlier studies provided evidence for G × G interactions between DENVs and *A. aegypti* (Lambrechts et al. 2009, 2013; Lambrechts 2011; Fansiri et al. 2013) as well as observations consistent with vector‐driven selection of DENVs (Hanley et al. 2008; Lambrechts et al. 2012; Quiner et al. 2014). Here, we hypothesized that DENV spatial distribution could result from local adaptation to mosquito vectors. We predicted that the spatial structure of DENV genetic diversity would match that of the mosquito vector populations. Indeed, *A. aegypti* consist of a patchwork of genetically differentiated populations at a fine geographical scale (Apostol et al. 1996; Garcia‐Franco et al. 2002; Huber et al. 2002; Bosio et al. 2005). Consistent with this expectation, for a different mosquito‐borne pathogen system, local adaptation of *Plasmodium* parasites to anopheline mosquito populations was shown at the vector species level (Joy et al. 2008).

We experimentally measured the extent of DENV adaptation to local *A. aegypti* populations using field‐collected viruses and mosquitoes from Thailand. We compared the vector competence of two wild‐type *A. aegypti* populations from Kamphaeng Phet and Bangkok for a large set of DENV‐1 isolates defined as sympatric or allopatric according to their geographical origin. Vector competence is the ability of a mosquito to acquire infection and eventually allow transmission of a pathogen after imbibing an infectious blood meal (Kramer and Ebel 2003). Kamphaeng Phet and Bangkok are located about 300 km apart. Strong genetic differentiation of *A. aegypti* populations was previously demonstrated between these two locations (Bosio et al. 2005). Under the hypothesis of parasite local adaptation, we expected vector competence to be higher in sympatric versus allopatric vector–virus combinations. Following the methods of Blanquart et al. (2013), we used the ‘sympatric versus allopatric’ contrast to measure local adaptation. According to these authors, a linear model describing the pattern of mean fitness as the sum of a habitat (i.e., mosquito population) effect, a genotype (i.e., virus isolate) effect, and a sympatric versus allopatric effect is the most powerful and straightforward way to detect local adaptation. This test is based on the residual variability when accounting for the effects of genotype and habitat.

## Materials and methods

### Ethics statement

The study protocol was approved by the Institutional Review Boards of the Thai Ministry of Public Health, Walter Reed Army Institute of Research, and University of California at Davis.

### Mosquitoes

Wild *A. aegypti* larvae and pupae were collected during 2009 (experiment 1) and 2011 (experiment 2) from multiple artificial containers in the Ladkrabang and Ratchathewi Districts, Bangkok and in the Muang District, Kamphaeng Phet Province, Thailand. No specific permission was required to conduct outdoor mosquito collections because they were carried out in public locations. Indoor collections were made with verbal permission of the homeowners. F_0_ adults were allowed to emerge in the laboratory, mate randomly, and feed on commercial defibrinated sheep blood (purchased from National Laboratory Animal Center, Mahidol University, Nakhon Pathom, Thailand) through a membrane feeding system. Institutional Animal Care and Use Committee approval was not required because sheep blood collection took place postmortem as a by‐product of a commercial enterprise. F_1_ eggs were collected on paper towel lining oviposition cups and stored under high humidity. Prior to experiments, eggs were hatched synchronously by placing them under low pressure for 30 min. Larvae were reared in 24 × 34 × 9 cm plastic trays filled with 2.0 L of dechlorinated tap water at a density of approximately 200 first instars per tray and fed a standard diet of approximately 1.0 g of fish food pellets (C.P. Hi Pro; Perfect Companion Group Co. Ltd, Bangkok, Thailand) per tray. After emergence, F_1_ adults were housed in plastic 30 × 30 × 30 cm cages (Megaview Science Education Service Co. Ltd, Taichung, Taiwan) with permanent access to 10% sucrose. They were maintained under insectary conditions at 28 ± 1°C, 80% humidity, and with a 12:12 hour light:dark cycle.

### Virus isolates

Fourteen DENV‐1 isolates (designated hereafter as B76, B88, B1, B2, B3, B4, B5, K15, K25, K1, K2, K3, K4, and K5 with B for Bangkok and K for Kamphaeng Phet) were chosen from frozen banked viral isolate seed stocks. The isolates are numbered by experiment, with a different numbering system in each experiment. All isolates were originally recovered from human serum samples obtained in 2009 during routine surveillance for diagnostic public health testing at AFRIMS from clinically ill patients with dengue attending Kamphaeng Phet Provincial Hospital (Kamphaeng Phet) and Queen Sirikit National Institute of Child Health (Bangkok), with the exception of isolates K1 and K2, which were derived from an approved research protocol. In all cases, informed consent of the patients was not necessary because viruses were previously isolated in laboratory cell culture (Klungthong et al. 2007) as the gold standard diagnostic procedure (unrelated to this study) and, therefore, were no longer considered human samples. No significant bias toward particular genotypes of the viral population should be expected among viruses isolated from clinically ill patients compared to mildly symptomatic or asymptomatic people (Duong et al. 2015). Each isolate was passaged four times in *A. albopictus* cells (C6/36, ATCC CRL‐1660) prior to its use for experimental infections of mosquitoes.

### Oral challenge

Experimental infections of mosquitoes were conducted as previously described (Lambrechts et al. 2012; Pongsiri et al. 2014). Briefly, two sets of 2‐day‐old confluent cultures of C6/36 cells in 25 cm^2^ flasks (approximately 10^7^ cells/flask) were inoculated with 1.0 mL of stock virus per flask and incubated at 35°C. Supernatant was harvested 5 and 6 days postinoculation to prepare the infectious blood meal of experimental blocks 1 and 2, respectively. The artificial blood meal consisted of a 1:1 mix of commercial defibrinated sheep blood and virus suspension. 2 to 7‐day‐old *A. aegypti* F_1_ females deprived of sucrose and water for 24 h were offered an infectious artificial blood meal for 30 min through pieces of desalted porcine intestine stretched over water‐jacketed glass feeders maintained at 37°C. Samples of the blood meals were saved prior to the artificial feeding to determine the infectious dose by subsequent titration in a plaque assay. After blood feeding, mosquitoes were briefly sedated with CO_2_ from dry ice and fully engorged females were transferred to clean paper cups. Unfed or partially fed females were discarded. Engorged females were maintained under standard insectary conditions, as described above, and provided cotton soaked with 10% sucrose *ad libitum*.

### Vector competence

Vector competence of the two *A. aegypti* populations for the 14 DENV‐1 isolates was evaluated 14 days after they imbibed the infectious blood meal. Upon harvest, the legs of each female were removed and placed individually in 1.0 mL of mosquito diluent (MD), consisting of RPMI 1640 medium with 10% heat‐inactivated FBS with 100 units/mL penicillin and 100 μg/mL streptomycin. Bodies were kept separately in 1.0 mL of MD. Samples were stored at −70°C before processing. Samples were quickly thawed in a water bath at 35 ± 2°C and homogenized in a mixer mill (Qiagen, Hilden, Germany) at 24 cycles/s for 2 min. Bodies were screened qualitatively by serotype‐specific RT‐PCR (experiment 1) or plaque assay (experiment 2). In experiment 1, total RNA was extracted from 140 μL of body homogenates using QIAamp viral RNA mini kit (Qiagen) according to the manufacturer's instructions. RT‐PCR was performed with 5 μL of extracted RNA following a standard protocol (Lanciotti et al. 1992) with the following modifications: (i) 1× PCR buffer II supplied with Amplitaq DNA Polymerase (Applied Biosystems, Waltham, MA, USA) was used instead of the standard buffer (50 mm KCl, 10 mm Tris pH 8.5 and 0.01 mm gelatin) in both the first round RT‐PCR and the second round PCR (nested PCR); (ii) the first round RT‐PCR reaction contained Avian myeloblastosis virus reverse transcriptase (Promega, Madison, WI, USA) instead of rav‐2 recombinant reverse transcriptase; (iii) the 1:50 dilution of the first round RT‐PCR product was used as the template in the nested PCR; (iv) the nested PCR reaction contained 12.5 pmol of each primer instead of 50 pmol; and (v) the number of the nested PCR cycles was increased from 20 cycles to 25 cycles (Lanciotti et al. 1992). Plaque assay was performed in rhesus monkey kidney cells (LLC‐MK_2_, ATCC #CCL‐7) as described previously (Thomas et al. 2009). Briefly, the homogenized samples were passed individually through a 0.22 μm syringe filter unit and 0.5 and 0.1 dilutions were prepared in MD. The samples were placed in an ice bath and 100 μL/well were inoculated into a monolayer of LLC‐MK_2_ cells in 24‐well plates. The virus was adsorbed for 1 h at room temperature (20–28°C) on a rocker platform. The inoculum was removed, and 0.5 mL/well of a first overlay of medium was added. The cells were incubated for 5 days at 35 ± 1°C in a 5 ± 0.5% CO_2_ incubator. The cells were stained with a second overlay of medium containing 4% neutral red (Sigma, St. Louis, MO, USA). Plaques were counted and plaque‐forming units (PFU)/mL were calculated. Although different isolates may have a differential ability to form plaques in LLC‐MK2 cells, this should not affect the ability to test for local adaptation in a full‐factorial design whereby each mosquito population is challenged with each virus isolate.

### Virus sequencing and phylogenetic analysis

Sequences of the viral envelope (*E*) gene of two of the isolates of the study (K15 and K25) were previously obtained and deposited to GenBank (accession numbers JN638326 and JN638327). For the remaining 12 isolates, viral RNA was extracted by QIAamp viral RNA mini kit (Qiagen) according to the manufacturer's instructions. DNA fragments of the *E* gene were synthesized and amplified using AccessQuick RT‐PCR System (Promega) per manufacturer's recommendations. The PCR‐amplified DNA fragments were purified using QIAquick PCR purification kit (Qiagen) before sending for Sanger sequencing by AITbiotech (Singapore). The 12 *E* gene sequences generated in this study were submitted to GenBank (accession numbers KT373891–KT373902). *E* gene sequences of the 14 isolates of the study were analyzed with 33 *E* gene sequences from GenBank representing the background DENV‐1 genetic diversity. A maximum likelihood (ML) tree was constructed with PhyML 3.0 (Guindon et al. 2010). The GTR+G model of nucleotide substitution was selected as the best‐fit model for the ML tree construction using the jModelTest (Posada 2008). Bootstrap resampling analysis was performed using 1000 replicates.

### Statistical analyses

The study was run in two separate experiments that used populations of *A. aegypti* that were sampled from the same locations, but during two different years. Within both experiments, the same batch of mosquitoes was exposed twice with the same set of DENV isolates on two successive days, that is, two experimental blocks. In the two blocks, viruses came from the same passage in cell culture, but were harvested 1 day apart, potentially resulting in small differences in infectious titer (ranging from 1.5 × 10^5^ PFU/mL to 8.5 × 10^6^ PFU/mL across isolates). Multivariate analyses of raw vector competence indices included the effects of blood meal titer (log_10_‐transformed), experiment, mosquito population, population × experiment interaction, viral isolate, and population × isolate interaction. When accounting for the blood meal titer, the block effect was always insignificant and was, therefore, removed from the model. The two experiments involved two different sets of DENV isolates. The isolate effect, therefore, was nested within the effect of the experiment. Midgut infection and viral dissemination (binary variables) were analyzed with logistic regressions, whereas the infectious titer in legs (continuous variable) was analyzed with an analysis of variance (anova) after log_10_‐transformation. Following recommendations in Blanquart et al. (2013), DENV local adaptation was measured by the sympatric versus allopatric contrast in a linear model describing variation in vector competence as a sum of a habitat (i.e., mosquito population) effect, a deme quality (i.e., isolate) effect, and a sympatric versus allopatric effect. First, the mean vector competence index by isolate was calculated for each experimental block. Second, mean vector competence indices were corrected for the effects of blood meal titer and experiment by taking the residuals from an anova that accounted for their respective marginal effects. Third, the mean residual of the two experimental blocks was calculated for each isolate and analyzed with an anova as a function of mosquito population, virus isolate, and allopatric versus sympatric contrast. The anova was weighted by the mean sample size (number of mosquitoes tested) by isolate. The *F* ratio was used to test whether the means of the two distributions (allopatric versus sympatric combinations) were significantly different from each other. All analyses were performed with the software JMP v10.0.2.

## Results

Vector competence was assessed in a total of 635 individual *A. aegypti* females (Table S1), of which 176 (27.7%) were tested in experiment 1 and 459 (72.3%) in experiment 2. Mosquitoes were exposed to 14 DENV‐1 isolates of which seven were isolated from Kamphaeng Phet (KPP) and seven from Bangkok (BKK). There were near equal proportions of individuals from the KPP mosquito population (50.9%) and from the BKK mosquito population (49.1%). Likewise, the proportion of mosquitoes exposed to the KPP isolates (50.9%) was similar to the proportion of mosquitoes exposed to the BKK isolates (49.1%). The dataset consisted of 51.7% allopatric pairings and 48.3% sympatric pairings, with sample sizes of 10–39 individuals per pairing (mean = 22.7; median = 22). Across experiments, experimental blocks and isolates, blood meal titers ranged from 1.5 × 10^5^ to 8.5 × 10^6^ PFU/mL (mean = 2.7 × 10^6^; median = 1.8 × 10^6^).

**Figure 1:**
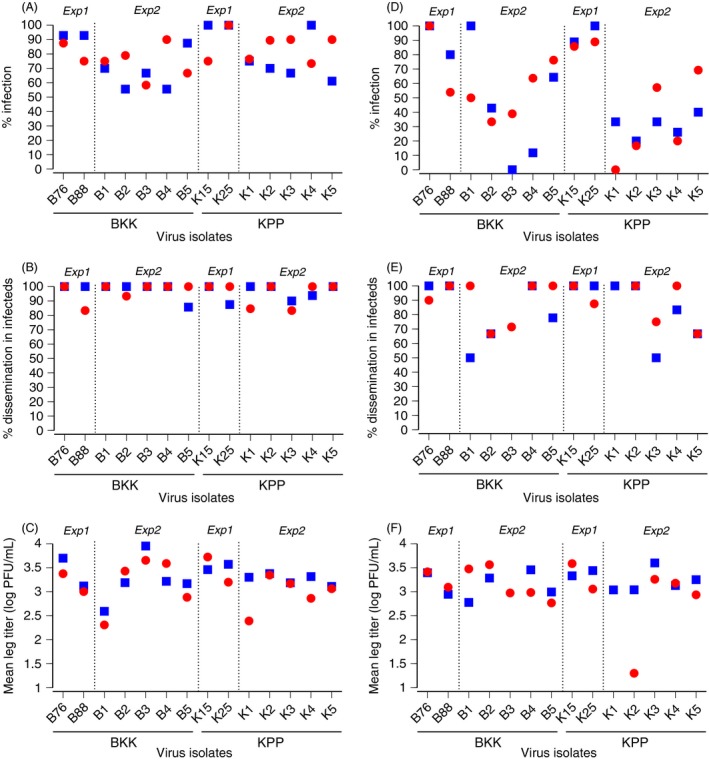
Vector competence indices. Graphs show the percentage of infected mosquitoes (A, D), percentage of infected mosquitoes with a disseminated infection (B, E), and mean infectious titer of disseminated virus (C, F) for each combination of the 14 DENV‐1 isolates (indicated along the *x*‐axis) and two *A. aegypti* populations (BKK: blue squares; KPP: red circles). Each population‐virus pair was tested in two experimental blocks (block 1: A–C; block 2: D–F). In the two experimental blocks, viruses came from the same passage in cell culture, but were harvested separately, which resulted in slightly different blood meal titers (ranging from 1.5 × 10^5^ PFU/mL to 8.5 × 10^6^ PFU/mL). Within each block, however, mosquitoes from both populations were exposed to the same blood meal titer. Dotted, vertical lines separate experiment 1 (*Exp1*) and experiment 2 (*Exp2*).

Overall, 67.4% of mosquitoes had a midgut infection, and 93.5% of infected mosquitoes had a disseminated infection. The percentage of infected mosquitoes varied substantially from 0% to 100% across population‐isolate pairings and experimental blocks (Fig. 1A), whereas the percentage of mosquitoes with a disseminated infection was more narrowly distributed above 80% (Fig. 1B). Leg titers of mosquitoes with a disseminated infection ranged from 1.0 × 10^1^ to 1.8 × 10^4^ PFU/mL (mean = 2.9 × 10^3^; median = 2.0 × 10^3^) and varied significantly among population‐isolate pairings and experimental blocks (Fig. 1C). Blood meal titer was the strongest predictor of all vector competence indices (Table 1). A population × isolate interaction significantly influenced midgut infection rates (Table 1). Isolate had a highly significant effect on dissemination and leg titers (Table 1).

**Table 1:** Multiway analysis of vector competence indices. The proportion of infected mosquitoes and the proportion of infected mosquitoes with a disseminated infection were analyzed with a logistic regression. The leg titer of mosquitoes with a disseminated infection was analyzed by analysis of variance after log_10_‐transformation

Variable	d.f.	Infection		Dissemination		Leg titer	
		L‐R *χ* ^2^	*P*‐value	L‐R *χ* ^2^	*P*‐value	*F* ratio	*P*‐value
Blood meal titer	1	71.2	<0.0001	13.9	0.0002	7.14	0.0079
Experiment	1	128	<0.0001	<0.001	0.9984	18.0	<0.0001
Mosquito population	1	1.90	0.1683	<0.001	0.9983	5.68	0.0177
Experiment × Population	1	8.30	0.0040	<0.001	0.9980	2.10	0.1481
Virus isolate (within Experiment)	12	8.57	0.7389	29.4	0.0034	4.74	<0.0001
Isolate × Population (within Experiment)	12	21.8	0.0403	17.3	0.1392	1.72	0.0616

L‐R, likelihood ratio; d.f., degrees of freedom.

**Figure 2:**
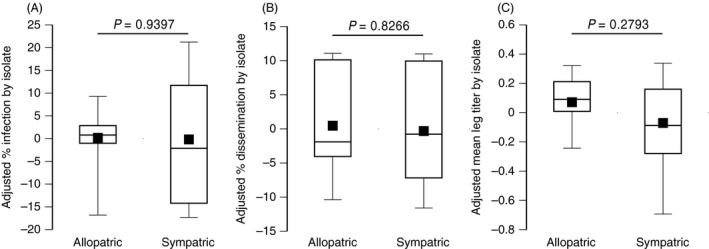
Tests of DENV local adaptation. Boxplots show the adjusted percentage of infected mosquitoes (A), adjusted percentage of infected mosquitoes with a disseminated infection (B), and adjusted mean infectious titer of disseminated virus (C) in allopatric versus sympatric combinations of the 14 DENV‐1 isolates and two *A. aegypti* populations of the study. Prior to testing for local adaptation, vector competence indices were adjusted for differences in blood meal titers and uncontrolled differences between the two experiments; values plotted are in arbitrary units. Each virus isolate is represented by the mean of two experimental blocks. *P*‐values above the bars indicate the statistical significance of the allopatric versus sympatric effect.

To test for DENV local adaptation, weighted mean vector competence indices were analyzed as a function of mosquito population, isolate, and sympatric versus allopatric contrast, after correcting for the effects of blood meal titer and experiment. The allopatric versus sympatric contrast did not significantly influence adjusted vector competence indices (Table 2; Fig. 2).

**Table 2:** Test statistics of DENV local adaptation. Analysis of variance (anova) of the mean proportion of infected mosquitoes, mean proportion of infected mosquitoes with a disseminated infection, and log_10_‐transformed leg titer by DENV isolate weighted by mean sample size. For each isolate, mean vector competence indices were adjusted for effects of blood meal titer and experiment, and averaged between the two experimental blocks prior to the anova

Variable	d.f.	Infection		Dissemination		Leg titer	
		*F* ratio	*P*‐value	*F* ratio	*P*‐value	*F* ratio	*P*‐value
Mosquito population	1	0.217	0.6496	0.304	0.5913	4.96	0.0459
Virus isolate	13	0.465	0.9071	1.81	0.1577	2.45	0.0653
Allopatric versus Sympatric	1	0.009	0.9268	0.055	0.8192	0.279	0.2790

d.f., degrees of freedom.

**Figure 3:**
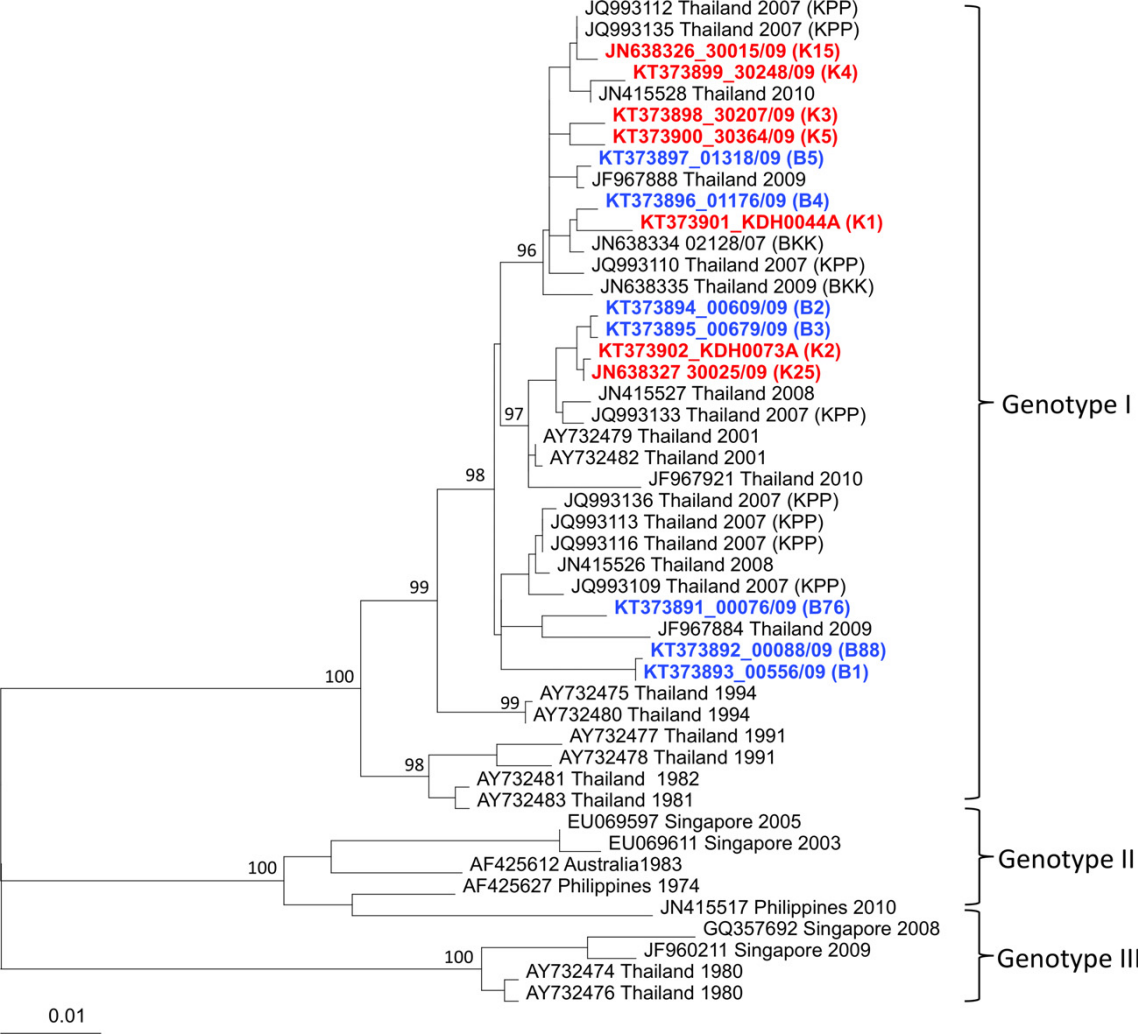
Phylogenetic relationships among DENV isolates. Maximum likelihood tree based on *E* gene sequences of 47 DENV‐1 isolates including 14 from this study (in color) and 33 from GenBank (in black font). Red and blue fonts indicate viruses isolated from patients in Kamphaeng Phet (KPP) and Bangkok (BKK), respectively. Bootstrap resampling values are shown at major nodes, and the scale bar indicates the number of base substitutions per site.

Phylogenetic analysis of the 14 viral isolates based on their *E* gene sequences revealed that they collectively belonged to three distinct clades within the genotype I of DENV‐1 (Fig. 3). Phylogenetic clustering did not correlate, however, with the geographical origin of the isolates; that is, BKK or KPP isolates were assigned to all three clades regardless of their location of origin. Although one clade only contained three BKK isolates, it also contained KPP isolates from other studies (Fig. 3). When vector competence was analyzed using the viral clade instead of the isolate, clade significantly influenced leg titers (*P *<* *0.001) but there was no significant population × clade interaction for any of the vector competence indices.

## Discussion

We compared vector competence indices of 14 low‐passage DENV isolates in two wild‐type *A. aegypti* populations that were collected either from the virus location of origin or from a distant location. We used a reciprocal cross‐infection design whereby each DENV isolate was tested in sympatric and allopatric mosquitoes. We hypothesized that vector competence would be higher on average in sympatric compared to allopatric pairs of mosquito and viruses, consistent with a pattern of virus local adaptation. Our analysis showed no evidence for local adaptation of DENV isolates to their mosquito vectors. It did not suggest local maladaptation either, because mosquitoes were not more resistant to sympatric than allopatric DENV isolates.

As for any negative result, absence of evidence in support of the local adaptation hypothesis does not rule out its existence. We tested local adaptation based on the sympatric versus allopatric contrast, which is considered the most powerful and straightforward way to detect local adaptation (Blanquart et al. 2013). A statistically significant population × isolate interaction effect on midgut infection rates indicated that the lack of local adaptation pattern in our study did not simply result from a lack of G × G interactions, a prerequisite for local adaptation (Lambrechts et al. 2009). The likelihood of midgut infection depended on the specific pairing of mosquito population and virus isolate, but sympatric pairings were not superior to allopatric pairings. Although a larger number of mosquito populations would be desirable to increase statistical power, our ability to detect G × G interactions indicates that power was sufficient to detect a statistical interaction. With only two mosquito populations, some forms of G × G interactions could drive a misleading pattern consistent with local adaptation that is independent of divergent selection. This was not the case in our study because despite significant G × G interactions, we did not observe a local adaptation pattern. Sampling dates of mosquitoes and viruses were temporally matched in experiment 1 (2009 viruses and 2009 mosquitoes), but not in experiment 2 (2009 viruses and 2011 mosquitoes). This could explain the observed experiment effect, and may have obscured detection of a local adaptation pattern.

Geographical variation in *A. aegypti* vector competence for reference DENV strains has been documented at scales ranging from local to continental (Gubler et al. 1979; Tardieux et al. 1990; Vazeille‐Falcoz et al. 1999; Bennett et al. 2002; Paupy et al. 2003; Lozano‐Fuentes et al. 2009). We chose to investigate local adaptation at a regional scale, based on study sites separated by about 300 km. The rationale was that DENV and *A. aegypti* populations are known to be genetically structured at a finer geographical scale than the sampling design we used. For example, phylogenetic analysis of DENV isolates from Kamphaeng Phet showed spatial structuring at the scale of primary school catchment areas within the province (Jarman et al. 2008). Likewise, *A. aegypti* populations in Kamphaeng Phet and Bangkok have been shown to be genetically differentiated based on mitochondrial DNA markers (Bosio et al. 2005). The genetic structure of virus and mosquito populations, however, is determined with genomic sequences or genetic markers that are not necessarily relevant to vector–virus interactions. In other words, virus and mosquito genetic determinants governing vector competence could be structured at a different geographical scale than neutral genetic markers that are typically used to infer population genetic structure. Our results revealed a population effect on vector competence phenotypes measured under standard environmental conditions, which confirmed that our two *A. aegypti* populations were genetically different with regard to vector competence for DENV‐1.

Local adaptation measures the match between adaptive genetic variation and environmental variation. However, local adaptation is not a necessary outcome of divergent selection because it can be hindered by gene flow (i.e., migration), confounded by genetic drift, and opposed by other selective forces (Kawecki and Ebert 2004). The lack of a local adaptation pattern, therefore, does not challenge earlier evidence for vector‐driven selection of DENV (Hanley et al. 2008; Lambrechts et al. 2012; Quiner et al. 2014). Multiple additional evolutionary forces likely act concomitantly to shape the observed genetic diversity of DENV populations. Previous analyses of DENV microevolution in Kamphaeng Phet suggested frequent immigration by human movement and demographic bottlenecks during transmission (Jarman et al. 2008; Rabaa et al. 2013). It is unlikely that *A. albopictus*, a secondary DENV vector found in Thailand, prevented local adaptation because *A. aegypti* is the predominant mosquito vector at our study sites (Chareonviriyaphap et al. 2003; Koenraadt et al. 2008).

Phylogenetic analysis revealed that our assignment of the DENV isolates to sympatric versus allopatric groups based on their geographical origin did not match their patterns of genetic divergence inferred from *E* gene sequences. Although, like for mosquitoes, nucleotide sequences used to infer phylogenetic relationships are not necessarily relevant to vector–virus interactions, this result indicates that the virus isolates we studied were not genetically structured at the regional scale that we investigated. Thus, high migration rates of viruses between BKK and KPP may sustain high levels of gene flow that hinder local adaptation (Kawecki and Ebert 2004). Migration provides one possible explanation to the lack of detectable local adaptation pattern in our study.

Local adaptation of mosquito‐borne pathogens to their vectors has only been examined in a handful of studies. Results from research with human malaria parasites support the hypothesis that the genetic structure of *Plasmodium* populations is driven by interactions with distinct populations of their mosquito vectors. Joy et al. (2008) reported that the population structure of *P. vivax* in Southern Mexico mirrors the distribution of two anopheline vector species. Based on experimental infections, they further demonstrated that parasites had superior infectivity in their sympatric mosquito species, consistent with local parasite adaptation (Joy et al. 2008). An experimental study on *P. falciparum* reported similar infection rates, but lower infection intensities in sympatric versus allopatric populations of *Anopheles coluzzii*, which was interpreted as a possible parasite adaptation to minimize the fitness cost to the vector (Harris et al. 2012). In addition to genetic interactions, local adaptation to environmental parameters such as temperature could also drive the distribution of vector and pathogen populations (Sternberg and Thomas 2014).

Our results do not support DENV adaptation to local *A. aegypti* populations as a major driver of the spatial distribution of DENV genetic diversity at the geographical scale that we examined. Vector‐driven selection might be counter‐acted by other evolutionary forces such as migration, genetic drift and human‐driven selection. Our phylogenetic analysis of DENV isolates was consistent with high levels of viral gene flow between study sites. The regional scale of this study might not be the most relevant geographical scale to test for DENV local adaptation to mosquitoes. An earlier study found that 57% of the total genetic variation within and between *A. aegypti* populations in Thailand, including at our study sites, was observed within collections (Bosio et al. 2005). Although parasites are predicted to adapt to locally common host genotypes (Lively and Dybdahl 2000), genetic heterogeneity of mosquito populations at each study site may have reduced a local adaptation signal. We cannot rule out, therefore, that DENV local adaptation occurs in places and times that we did not test or at a different geographical scale than the regional scale considered in this study, either at a smaller (local) or larger (continental) scale. Additional studies are needed to refine understanding of the complex evolutionary forces that shape DENV microevolution.

## Data archiving statement

Raw data for this study are provided in the supporting information.

## Supporting information


**Table S1.** Raw vector competence data.
